# Blood Pressure Level and Risk of Cardiovascular Disease Mortality in Patients With Kidney Failure Receiving Maintenance Peritoneal Dialysis: A Subanalysis

**DOI:** 10.1016/j.xkme.2026.101523

**Published:** 2026-09-10

**Authors:** Viangkaeo Lee, Haleh Siami, Vanessa-Giselle Peschard, Emily Huang, Mohsen Malekinejad, Chi-yuan Hsu

**Affiliations:** 1 University of California, San Francisco, CA; 2 Islamic Azad University of Medical Sciences, Tehran, Iran; 3 Biogen, South San Francisco, CA; 4 Bates College, Lewiston, ME

To the Editor:

Bezerra et al^1^ recently published a systematic review and meta-analysis, which examined the association between blood pressure (BP) and cardiovascular disease (CVD) in patients with kidney failure receiving maintenance peritoneal dialysis. This motivated us to conduct a subanalysis of our recently published systematic review and meta-analysis^2^ focusing on CVD because there may be a stronger association between elevated BP levels and CVD deaths than with deaths from other causes.

Three authors (EH, HS, and VL) independently determined the studies included in our systematic review^2^ that also reported CVD deaths; 2 authors (EH and HS) abstracted data regarding the number of CVD deaths as well as the effect sizes and 95% confidence intervals (CI); and 2 others (VL and V-GP) classified the CVD death ascertainment method (Item S1). Discrepancies were resolved through discussion. In one instance in which the reported lower bounds of the CI exceeded the parameter estimate,^3^ we reached out to the study author for clarification (but did not receive a response).

We used the same criteria as our original study^2^ to determine the studies that could be subsequently meta-analyzed. We excluded studies that only analyzed mean arterial pressure or that assumed a linear relation between BP level and adverse outcomes. Owing to concerns regarding reverse causation, we only included studies that examined the association between baseline BP level (not time-updated or average BP) and subsequent risk of CVD deaths. We used the same process (including pooling within-study estimates first) to accommodate for the fact that different studies used different cutoffs to divide systolic BP (SBP) (or diastolic BP) into categories.^2^ We again considered hazard ratios to be risk ratios (RR) owing to the rarity of the outcome.^2^

For SBP, we were able to perform meta-analysis for high (>140 mm Hg) and low (<100 mm Hg) categories, using SBP 100-140 mm Hg as the control. We were also able to perform meta-analysis for presence versus absence of hypertension. Between-study variance (τ^2^) was estimated using restricted maximum likelihood within a random-effects meta-analysis framework. Because there was only one study that met diastolic BP meta-analysis criteria, it could not be combined with any other study.

We identified 12 studies previously included in our systematic review that reported CVD deaths (Table 1). Further details regarding these studies, such as patient characteristics and follow-up duration, were discussed in our original study.^2^

**Table 1:** Summary of Included Studies Assessing BP and CVD Mortality Outcomes in Maintenance Peritoneal Dialysis Patients

Reference	Study Design (No. of Participants)	Total No. of CVD Deaths Out of All-Cause Deaths (% CVD Deaths)	CVD Death Ascertainment Method	Type of BP	Control Condition as Defined by Study Authors (mm Hg)	Risk Factor (mm Hg)	BP Variable Type (Static Refers to BP Ascertained at Baseline, and Not Updated or Averaged With Later BP Readings)	Effect Size^a^ as Reported by Study Authors (95% CI)
Bao et al (2019)^3^	Retrospective cohort (181)	42/102 (41%)	Investigator-defined classification—details not specified	SBP	Not applicable	Per 1-mm Hg increase	Continuous, static	HR = 1.00 (1.99-1.01) (not adjusted)
				DBP	Not applicable	Per 1-mm Hg increase	Continuous, static	HR = 0.98 (0.96-0.99) (not adjusted)
Beduschi et al (2015)^4^	Retrospective cohort (2,890)	247/550 (45%)	Investigator-defined classification—details not specified	HTN vs no HTN	No HTN	HTN	Dichotomous, static	HR = 0.99 (0.73-1.35)^b^
								Subdistribution HR = 1.04 (0.76-1.42)^b^ Fine and Gray competing risk model
Chen et al (2022)^5^	Prospective cohort (216)	22/56 (39%)	Investigator-defined classification—details not specified	MAP	Not applicable	Per 1-mm Hg increase	Continuous, static	HR = 1.022 (0.976-1.071) (not adjusted)
Dai et al (2020)^6^	Prospective cohort (260)	33/64 (52%)	Investigator-defined classification—details not specified	SBP	Not applicable	Per 1-mm Hg increase	Continuous, static	HR = 1.023 (1.007-1.040)
				DBP	Not applicable	Per 1-mm Hg increase	Continuous, static	HR = 1.008 (0.979-1.038) (not adjusted)
Fang et al (2009)^7^	Retrospective cohort (306)	35/74 (47%)	Clinical chart review	SBP	Not applicable	Per 1-mm Hg increase	Continuous, static	HR = 0.971 (0.943-0.999)
Goldfarb-Rumyantzev et al (2005)^8^	Retrospective cohort (1,053)	339 (no. of all-cause deaths not reported)	Registry-based coding	SBP	111-120	≤100	Ordinal, static	HR = 3.83 (1.93-7.59)^b^
						101-110	Ordinal, static	HR = 2.92 (1.51-5.67)^b^
						121-130	Ordinal, static	HR = 1.42 (0.84-2.41)^b^
						131-140	Ordinal, static	HR = 1.15 (0.68-1.96)^b^
						141-160	Ordinal, static	HR = 1.04 (0.63-1.71)^b^
						161-180	Ordinal, static	HR = 1.40 (0.79-2.49)^b^
						>180	Ordinal, static	HR = 0.76 (0.32-1.82)^b^
				DBP	76-80	≤65	Ordinal, static	HR = 1.01 (0.64-1.58)^b^
						66-75	Ordinal, static	HR = 1.02 (0.71-1.46)^b^
						81-85	Ordinal, static	HR = 0.94 (0.63-1.40)^b^
						86-90	Ordinal, static	HR = 0.89 (0.56-1.41)^b^
						91-100	Ordinal, static	HR = 0.95 (0.58-1.55)^b^
						101-110	Ordinal, static	HR = 1.03 (0.44-2.41)^b^
						>110	Ordinal, static	HR = 2.82 (0.80-9.87)^b^
Liao et al (2011)^9^	Retrospective cohort (280)	37/77 (48%)	Investigator-defined classification—based on primary care physician documentation	HTN vs no HTN	No HTN	HTN	Dichotomous, static	HR = 1.638 (0.380-7.067) (not adjusted)^b^
Lyu et al (2019)^10^	Retrospective cohort (1,737)	95/208 (46%)	Investigator-defined classification—details not specified	MAP	96-119	≤95	Ordinal, static	HR = 1.08 (0.65-1.79)
						≥120	Ordinal, static	HR = 2.55 (1.38-4.70)
Qiu et al (2020)^11^	Retrospective cohort (1,656)	252/507 (50%)	Investigator-defined classification—details not specified	SBP	110-130	<110	Ordinal, static	HR = 1.66 (1.09-2.53)^b^
						>130	Ordinal, static	HR = 1.50 (1.09-2.09)^b^
					Not applicable	Per 5-mm Hg increase	Continuous, static	HR = 1.01 (0.98-1.04)
				DBP	Not applicable	Per 5-mm Hg increase	Continuous, static	HR = 1.03 (0.98-1.08)
				MAP	Not applicable	Per 5-mm Hg increase	Continuous, static	HR = 1.03 (0.98-1.07)
Wu et al (2023)^12^	Retrospective cohort (3,073)	286/571 (50%)	Death certificate for deaths in the hospital; expert consensus agreement for deaths outside of the hospital	HTN vs no HTN	No HTN	HTN	Dichotomous, static	HR = 1.86 (1.36-2.54)^b^
							Dichotomous, static	HR = 1.30 (1.08-1.79)^b^ Fine and Gray competing risk model
Xie et al (2020)^13^	Retrospective cohort (7,335)	537/1281 (42%)	Investigator-defined classification—details not specified	SBP	119-141	<119	Ordinal, static	HR = 1.37 (1.01-1.87)
							Ordinal, static	HR = 1.40 (1.02-1.93) Fine and Gray competing risk model
						>141	Ordinal, static	HR = 1.31 (1.09-1.58)^b^
							Ordinal, static	HR = 1.30 (1.08-1.56)^b^ Fine and Gray competing risk model
				DBP	68-85	<67	Ordinal, static	HR = 1.53 (1.13-2.07)
							Ordinal, static	HR = 1.55 (1.13-2.12) Fine and Gray competing risk model
						>85	Ordinal, static	HR = 1.22 (1.01-1.48)
							Ordinal, static	HR =1.18 (0.97-1.43) Fine and Gray competing risk model
				MAP	89-102	<88	Ordinal, static	HR = 1.36 (1.02-1.82)
							Ordinal, static	HR = 1.39 (1.03-1.86) Fine and Gray competing risk model
						>102	Ordinal, static	HR = 1.26 (1.05-1.52)
							Ordinal, static	HR = 1.25 (1.03-1.51) Fine and Gray competing risk model
Xu et al (2021)^14^	Retrospective cohort (376)	37/70 (53%)	Determined by clinical team	SBP	Not applicable	Per 1-mm Hg increase	Continuous, static	HR = 0.975 (0.953-0.997)
							Continuous, static	HR = 1.000 (0.998-1.003) (not adjusted)
				DBP	Not applicable	Per 1-mm Hg increase	Continuous, static	HR = 1.013 (0.984-1.043)

Abbreviations: BP, blood pressure; CI, confidence inetrval; CVD, cardiovascular disease; DBP, diastolic BP; HTN, hypertension; MAP, mean arterial pressure; SBP, systolic BP.

a Cox models unless otherwise specified below.

b Included in the meta-analysis.

Approximately half of the deaths were due to CVD (Table 1).

Compared with all-cause mortality, CVD mortality in general had a stronger association with elevated BP levels. For example, the RR for SBP > 140 mm Hg versus 100-140 mm Hg was 1.31 (95% CI 1.13-1.51) for CVD death (Fig 1A), greater than the RR of 1.15 (0.99-1.34) for all-cause death.^2^

**Figure 1:**
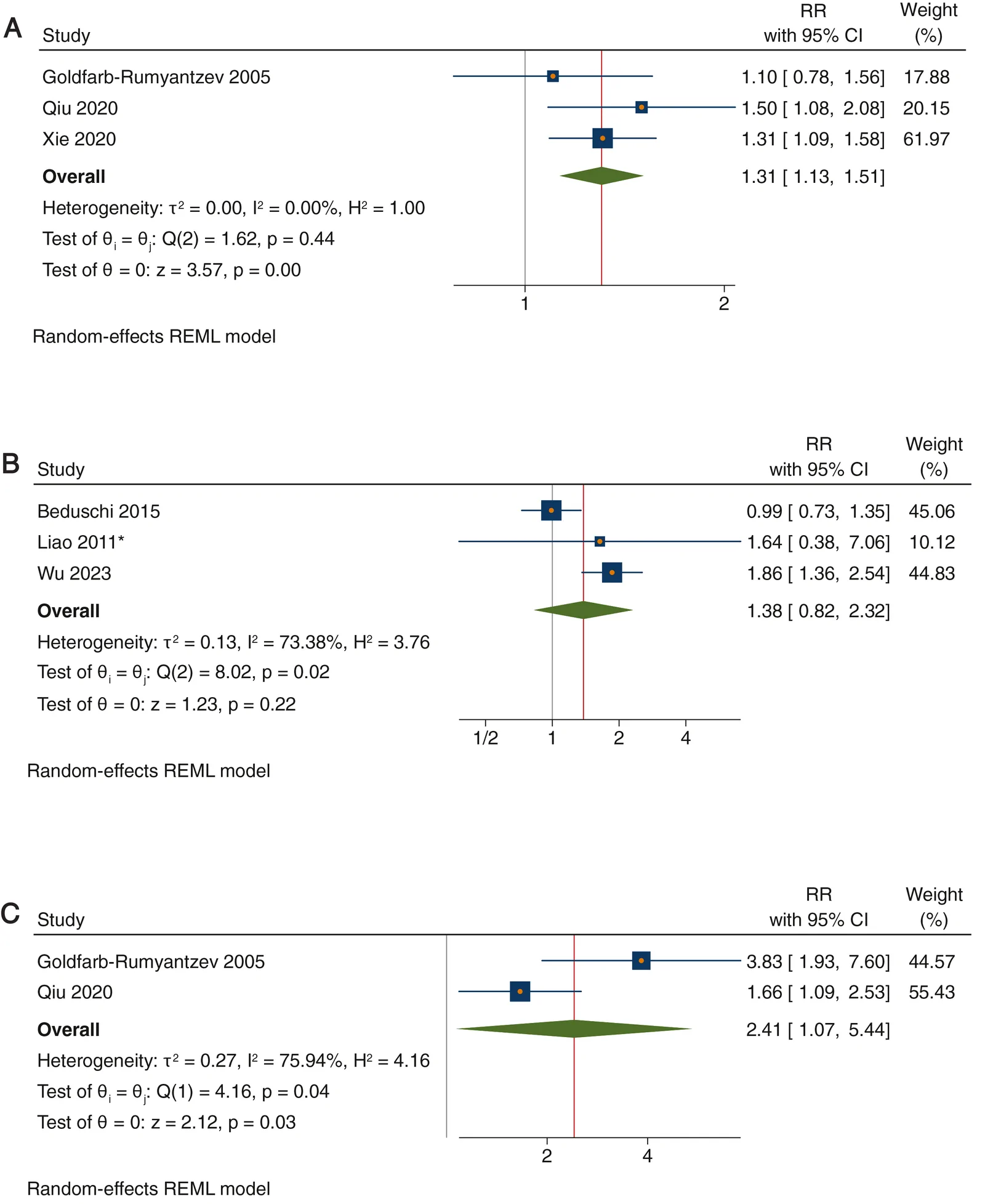
Forest plots of risk ratios (RR) for blood pressure and outcome of cardiovascular disease mortality among patients with kidney failure receiving maintenance peritoneal dialysis. (A) High systolic blood pressure (>140 mm Hg) versus control (100-140 mm Hg). Similar results were seen when the Xie 2020 Fine and Gray model estimate was used. Sensitivity analysis using leave-one-out meta-analysis yielded point estimates (RR) ranging from 1.26-1.35. (B) Presence versus absence of hypertension. Similar results were seen when the Beduschi 2015 or Wu 2023 Fine and Gray model estimate was used. Sensitivity analysis using leave-one-out meta-analysis yielded point estimates (RR) ranging from 1.01-1.85. ∗Liao 2011 was unadjusted. (C) Low systolic blood pressure (<100 mm Hg) versus control (100-140 mm Hg). Sensitivity analysis using leave-one-out meta-analysis was not performed as there were only 2 studies here. Abbreviations: CI, confidence interval; REML, restricted maximum likelihood.

Although the RR for presence versus absence of hypertension was 1.38 (0.82-2.32) for CVD death (Fig 1B), less than the RR of 1.75 (0.91-3.35) for all-cause death in our original study,^2^ this appeared to be a function of the studies that were included in the meta-analyses. For each individual study, the RR was higher for CVD deaths than for all-cause deaths (eg, 0.99 vs 0.98 for Beduschi et al,^4^ 1.64 vs 0.76 for Liao et al,^9^ and 1.86 vs 1.83 for Wu et al^12^).

At the same time, CVD mortality was also more strongly associated with SBP < 100 mm Hg versus 100-140 mm Hg, with RR 2.41 (95% CI 1.07-5.44) (Fig 1C) compared with 1.76 (95% CI 1.36-2.29) in our original study.^2^

Recent studies by our group^2^ and Bezerra et al^1^ are the first to our knowledge that systematically reviewed the observational studies regarding how level of BP is related to future risk of adverse events in patients with kidney failure receiving maintenance peritoneal dialysis. We screened 3,546 nonduplicate records and included 30 studies.^2^ Bezerra et al^1^ screened 852 and included 24 studies. A limitation in both the meta-analyses was heterogeneity, including inconsistencies in how cause of death was ascertained. However, we arrived at similar conclusions as follows: SBP exhibited a U-shaped association with all-cause mortality.

The exact reason for the U-shaped association between SBP and all-cause and CVD mortality in this patient population is not known. It is interesting that Georgianos et al^15^ reported, among 108 Greek patients, that higher quartiles of 24-hour ambulatory SBP monitoring were associated with stepwise higher risks of myocardial infarction, stroke, and all-cause death, but no such associations were observed for increasing quartiles of routinely measured office SBP (our original systematic review^2^ did not include Georgianos et al^15^ because this study did not report all-cause mortality as a standalone outcome).

Thus, conducting larger studies in different geographic regions of the world using ambulatory BP monitoring and comparing readings with home and office BP measurements in patients with kidney failure receiving maintenance peritoneal dialysis may be one promising avenue of research going forward.

## Supplementary Materials

Supplementary File (PDF) Figure S1; Item S1.
