# Supplementary material for: Blood Pressure Level and Risk of Cardiovascular Disease Mortality in Patients With Kidney Failure Receiving Maintenance Peritoneal Dialysis: A Subanalysis
Source: Kidney Med. 2026 Sep 10;8(12):101523. doi: 10.1016/j.xkme.2026.101523 (PMC13756250; doi:10.1016/j.xkme.2026.101523)
Supplement: Supplementary file 1 [file mmc1.pdf]

SUPPLEMENTARY MATERIAL

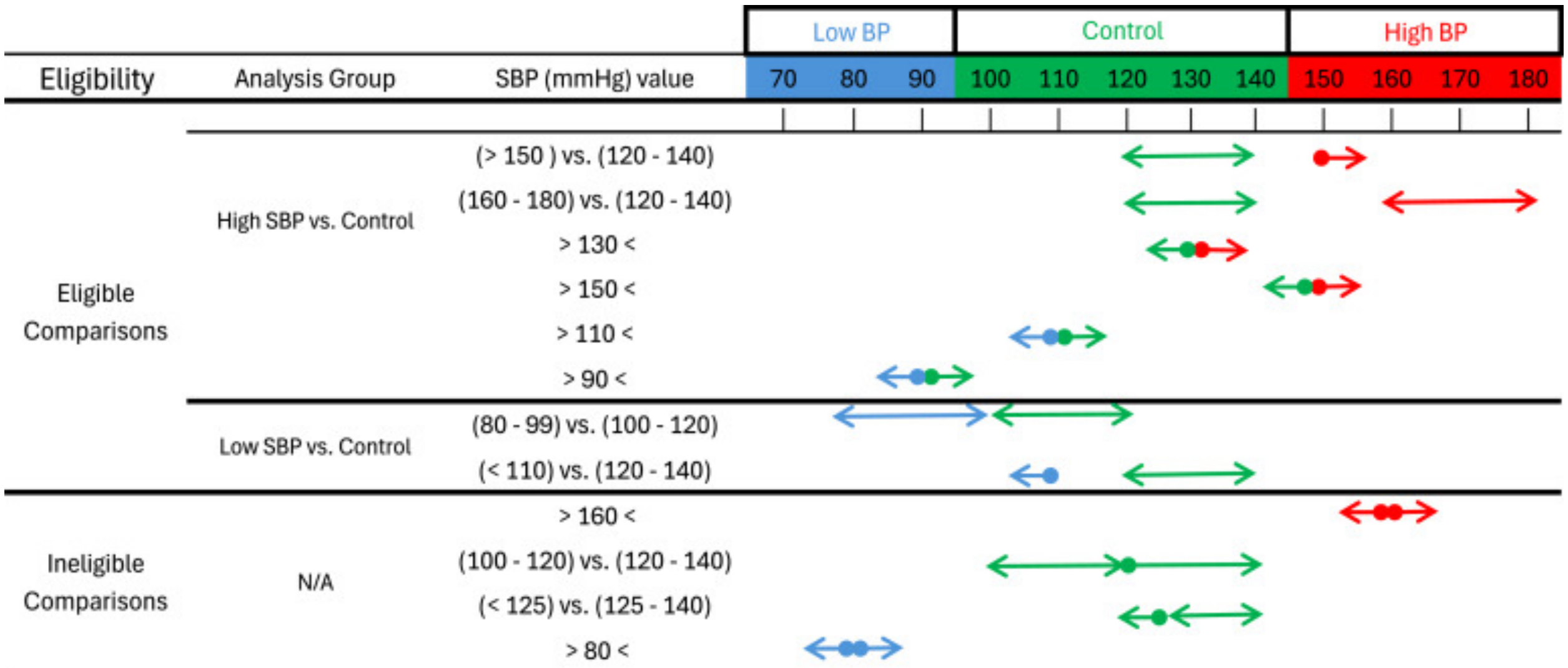

Figure S1. Eligibility framework for inclusion of systolic blood pressure (SBP) comparisons in the meta-analysis. The figure outlines the criteria used to determine whether SBP comparisons from individual studies qualified for pooled analysis. The predefined reference SBP interval was 100-140 mm Hg, whereas comparison groups outside this interval were categorized as exposure groups representing lower or higher BP levels. Studies were considered eligible when at least 1 comparison boundary fell within ±10 mm Hg of the reference interval (90-110 mm Hg for the lower threshold and 130-150 mm Hg for the upper threshold). Green arrows denote SBP categories within the predefined reference range of 100-140 mm Hg. Blue arrows represent SBP groups below the reference interval by more than 10 mm Hg, whereas red arrows indicate groups exceeding the upper boundary by more than 10 mm Hg. Eligible comparisons were required to include both a reference category (green) and an exposure category (blue or red). The comparison examples shown were adapted from studies included in the meta-analysis and are presented for illustrative purposes only. Abbreviations: BP, blood pressure; N/A, not applicable.

Item S1. Supplementary Methods

Systematic Review Eligibility Criteria

Study Population
Among the 30 studies included in our original systematic review and meta-analysis (Lee V, et al. Kidney Med. 8(2):101193, Published online November 26, 2025), we focused on the 12 studies which reported cardiovascular disease (CVD) deaths in this sub-analysis.

Exposure
The exposure of interest was blood pressure (BP).

Comparators
Eligible studies compared different BP levels (systolic and diastolic) and the presence versus absence of hypertension.

Outcome
The primary endpoint was cardiovascular disease (CVD) mortality.

Criteria for Meta-analysis Inclusion

Separate meta-analyses were performed according to BP exposure type: (1) systolic BP (SBP), (2) diastolic BP (DBP), and (3) hypertension status. Hypertension definitions varied by study and generally incorporated BP thresholds and/or antihypertensive medication use.

Studies assuming a strictly linear association between BP and mortality risk (e.g., risk increase per 10-mm Hg rise in SBP alone) were excluded because available evidence suggested a nonlinear relationship between BP and mortality, with elevated risk observed at both low and high SBP levels.

Most studies assessed baseline BP in relation to subsequent mortality risk. A small number treated BP as a time-dependent variable or used average BP measurements collected over time. To minimize potential bias associated with those approaches, only studies examining baseline BP values in relation to future all-cause mortality were included in the pooled analyses.

SBP Analysis

SBP served as the primary exposure because it remains the principal therapeutic target in modern hypertension trials. The reference SBP category was defined as 100 to 140 mm Hg, whereas SBP values above this range were considered exposure categories.

Because reporting methods differed across studies, predefined criteria were developed for study inclusion. SBP ranges falling within 100 to 140 mm Hg were considered sufficiently comparable for pooling. Some studies used single-threshold cutoffs instead of comparing bounded ranges. Such comparisons were eligible if cutoff values were within ±10 mm Hg of the predefined reference range boundaries. Comparisons using more extreme thresholds outside these margins were excluded. Studies comparing groups entirely within the reference range were also excluded.

These decision rules are illustrated in Figure S1. Arrows shown in green denote comparison groups with SBP values that fall within the predefined reference range of 100-140 mm Hg used for the meta-analysis. Blue arrows indicate SBP groups below the reference range by more than 10 mm Hg, whereas red arrows represent groups exceeding the upper boundary by more than 10 mm Hg. To qualify for inclusion, a comparison was required to contain both a reference group (green) and an exposure group (blue or red).

We also evaluated the association between lower SBP levels and all-cause mortality while retaining 100-140 mm Hg as the reference category.

Hypertension Status Analysis

Several studies classified participants according to the presence or absence of hypertension, usually based on BP measurements together with antihypertensive medication use, as defined within individual studies.

Statistical Analysis

Meta-analyses were conducted using Stata version 17. Pooled summary estimates and corresponding 95% confidence intervals (CIs) were generated using the “metan” command. Statistical heterogeneity across studies was evaluated using Cochran’s Q test and the I^2 statistic, with a significance threshold of $\alpha = 0.05$. Between-study variance (τ^2) was estimated using restricted maximum likelihood (REML) within a random-effects meta-analysis framework.

Random-effects models were selected a priori because of anticipated clinical, demographic, and methodological heterogeneity among studies in addition to statistical

variability. When studies contributed multiple eligible but mutually exclusive comparisons (e.g. Goldfarb-Rumyantzev 2005), estimates within the same study were first pooled using a fixed-effect approach to generate a single study-level estimate before inclusion in the overall meta-analysis.

When both adjusted and unadjusted estimates were available, the most comprehensive multivariable-adjusted models were preferentially included. Most studies used Cox proportional hazards regression models. A minority additionally reported Fine and Gray competing-risk analyses that yielded similar estimates; in these cases, Cox model estimates were selected to maintain consistency across pooled analyses.

We also performed leave-one-out sensitivity analyses to assess pooled estimate ranges after sequential exclusion of individual studies.
